# Effects of *Withania somnifera* (Ashwagandha) Supplementation on Exercise Performance: A Systematic Review and Three-Level Meta-Analysis

**DOI:** 10.3390/nu18121915

**Published:** 2026-06-12

**Authors:** Xiupeng Li, Hansen Li, Shuqi Yao, Ying Hou, Aiping Chi

**Affiliations:** 1 School of Physical Education, Shaanxi Normal University, Xi’an 710119, China; lixiupeng_0911@yeah.net (X.L.); 2023300314@snnu.edu.cn (S.Y.); hy091922@snnu.edu.cn (Y.H.); 2 School of Physical Education, Sichuan Agricultural University, Ya’an 625014, China; hanson-swu@foxmail.com

**Keywords:** *Withania somnifera*, ashwagandha, exercise performance, sports nutrition, ergogenic aid, meta-analysis

## Abstract

Background/Objectives: Evidence for herbal ergogenic aids remains uncertain, and ashwagandha trials span heterogeneous performance domains. This review evaluated oral *Withania somnifera* supplementation on exercise performance and explored participant-, outcome-, formulation-, and supplementation-related moderators. Methods: PubMed, Web of Science, Cochrane Library, Embase, and SPORTDiscus-EBSCO were searched from inception to 1 April 2026. Eligible randomized controlled trials compared oral ashwagandha with placebo or control conditions and reported objective exercise-performance outcomes. Dependent effects were synthesized using restricted-maximum-likelihood three-level random-effects models; 95% prediction intervals, GRADE certainty ratings, subgroup analyses, and dose/duration meta-regressions were reported. Results: Thirteen trials involving 599 participants contributed 79 effect sizes. Samples were mainly young adults or athletes; reported ages included one 18–40-year trial and one late-adolescent athlete cohort aged 17.4 ± 1.7 years. Trial-level sex composition was four male-only, one female-only, three mixed-sex, and five incompletely reported cohorts. Ashwagandha improved overall exercise performance on average (Hedges’ g = 0.47, 95% CI [0.25, 0.69], *p* < 0.001; I^2^ = 60%; 95% prediction interval [−0.40, 1.33]), but the prediction interval crossed zero. Exercise type was the clearest moderator (P_between = 0.006): evidence was most consistent for aerobic endurance (g = 0.54, 95% CI [0.22, 0.85], *p* = 0.002), whereas strength effects were positive but uncertain and power or muscular endurance evidence remained sparse. Dose analyses were hypothesis-generating; 500–600 mg/day was the most evidence-supported extract-dose range. Conclusions: Oral ashwagandha may improve selected exercise-performance outcomes, particularly aerobic endurance, but benefits are not uniform across contexts. Future trials should be preregistered, adequately powered, double-blind, formulation-standardized, sex-stratified, and include rigorous blinding checks, mechanistic endpoints, adverse-event monitoring, and sport-specific performance tests.

## 1. Introduction

Exercise performance is determined by the interaction of training stimuli, nutritional support, recovery management, sleep quality, and psychological regulation rather than by training load alone [1,2,3,4]. Although greater training stimuli may promote adaptation, poorly matched load and recovery can increase accumulated fatigue, compromise immune function, and elevate injury risk. Accordingly, safe and practically feasible nutritional strategies that support training adaptation have become a central focus in sports nutrition and sports medicine [1,2,3,4]. Supplements such as caffeine, creatine, protein, and beta-alanine have relatively well-characterized ergogenic mechanisms, including central nervous system stimulation, phosphagen energy provision, muscle protein synthesis, and intramuscular buffering [5,6,7,8]. By contrast, plant-derived bioactive supplements are often characterized by chronic, multi-target, and adaptogenic actions that may influence stress regulation, inflammation, oxidative stress, sleep, and recovery quality.

*Withania somnifera*, commonly known as ashwagandha or Indian ginseng, is a traditional Ayurvedic herb that has attracted growing interest as a sports-nutrition supplement [9,10,11,12,13]. Ashwagandha contains alkaloids, flavonoids, polyphenols, glycosides, and steroidal lactones, with withanolides considered among the principal bioactive constituents [14,15,16]. Experimental and clinical studies suggest that ashwagandha may influence stress responses, oxidative balance, inflammation, immune function, endocrine status, sleep, and metabolism [12,13,15,16,17,18,19,20,21]. These actions provide biologically plausible pathways through which chronic supplementation could support exercise performance, especially when performance is constrained by recovery capacity, fatigue tolerance, or training adaptation.

The ergogenic profile of ashwagandha is unlikely to resemble that of acute stimulants. Its potential benefit is more plausibly mediated by chronic modulation of the physiological context in which training occurs. For example, effects on hypothalamic–pituitary–adrenal axis activity and cortisol responses may improve recovery conditions, whereas effects on sleep quality, perceived fatigue, anxiety, and psychological readiness may help athletes maintain training quality [17,18,19,20,21]. Anti-inflammatory and antioxidant actions may also attenuate excessive exercise-induced muscle damage or oxidative stress, and possible influences on mitochondrial function, oxygen transport, and energy metabolism may be relevant to endurance performance [15,16,22,23].

Randomized controlled trials have reported mixed findings. Some trials have observed improvements in maximal or peak oxygen uptake, time to exhaustion, muscular strength, and perceived recovery, whereas others have not shown clear benefits for power output, muscular endurance, or short-duration high-intensity performance. These inconsistencies may arise from differences in sex, training status, baseline fitness, dosage, intervention duration, testing protocols, and product standardization [22,23]. Because ashwagandha is a botanical extract, studies may also differ in plant part, extraction procedure, withanolide content, and actual active-constituent exposure [13,14,15,16].

Previous meta-analyses have provided important initial evidence but have several limitations [22,23]. First, some reviews emphasized a narrow range of outcomes, particularly VO_2_max, and did not adequately distinguish aerobic endurance, strength, power, muscular endurance, and recovery-related performance. Second, individual trials often report several outcomes from the same participants; treating these effect sizes as independent in conventional two-level meta-analysis can underestimate uncertainty. Third, potential moderators such as dosage, intervention duration, sex, training status, study design, and exercise type require more systematic evaluation. Therefore, the present systematic review and three-level meta-analysis were designed to retain multiple related effect sizes while explicitly accounting for their dependence. The objectives were to quantify the effect of oral ashwagandha supplementation on overall exercise performance, identify the performance domains most likely to respond, and assess the robustness and certainty of the available evidence.

## 2. Materials and Methods

### 2.1. Protocol and Reporting Standards

This systematic review followed the PRISMA 2020 statement [24]. The review protocol was registered with PROSPERO (registration number: CRD420261389980). Registration occurred after pilot testing of the extraction form and preliminary appraisal of the literature, but before final eligibility decisions and analyses; this timing is stated to maximize transparency and reduce selective reporting concerns. The PRISMA 2020 checklist is provided in Supplementary File S1.

### 2.2. Eligibility Criteria

Eligible studies were full-text randomized controlled trials published in peer-reviewed journals that examined oral ashwagandha supplementation, including root powder, root extract, standardized root extract, or a single-ingredient supplement in which *Withania somnifera* was the primary active component. Eligible comparators were placebo, inactive control, or control conditions matched for training and testing procedures. Participants were healthy late-adolescent athletes, adults, recreationally active individuals, trained individuals, or athletes. No restrictions were applied to sex, sport discipline, or training level. Studies had to report at least one objectively measured exercise-performance outcome with sufficient data to calculate or extract a standardized effect size.

Because ashwagandha is generally investigated as a chronic supplementation strategy rather than as an acute ergogenic aid, no post-ingestion testing window was applied. Eligible interventions had a clearly defined supplementation period. Across included trials, intervention durations mainly ranged from 2 to 12 weeks, and the most frequently used dosages were 500, 600, and 1000 mg/day [25].

Studies were excluded if they were animal, in vitro, or mechanistic cell studies; non-randomized intervention studies; cross-sectional studies; case reports; single-arm pre–post studies; reviews; meta-analyses; registry-only records; conference abstracts; dissertations; or reports without retrievable full text. Studies were also excluded when they used multi-ingredient formulations or co-interventions from which the independent effect of ashwagandha could not be isolated; did not include exercise-performance outcomes; reported only non-performance outcomes such as sleep, fatigue, hormonal markers, biochemical markers, or body composition; provided insufficient data after author contact, supplementary material review, and figure extraction; or clearly overlapped with another report from the same sample. When multiple reports were available for the same study, the report with the most complete sample and performance data was prioritized. Both single- and double-blind trials were eligible, and blinding status was examined as a moderator.

### 2.3. Information Sources and Search Strategy

PubMed, Web of Science, Cochrane Library, Embase, and SPORTDiscus via EBSCOhost were searched from database inception to 1 April 2026. Search terms combined ashwagandha-related terms with terms for exercise performance and physiological performance domains. The core Boolean strategy was (ashwagandha OR “*Withania somnifera*” OR “Indian ginseng” OR “winter cherry” OR Withania) AND (“exercise performance” OR “physical performance” OR “physical capacity” OR “exercise capacity” OR “aerobic capacity” OR “aerobic fitness” OR “cardiorespiratory fitness” OR “endurance performance” OR “anaerobic performance” OR “muscular strength” OR “muscular endurance” OR “power output” OR VO_2_max OR “time to exhaustion” OR “jump performance” OR “explosive performance” OR “strength performance”). No publication year restrictions and no automated database filters were applied. Only full-text original studies published in English were eligible to ensure consistency in screening and data extraction. Reference lists of included studies and relevant reviews were screened manually to identify additional eligible studies. Articles were retained for full-text assessment when eligibility could not be determined from the title or abstract. Database-specific search strategies are reported in the Supplementary File S2.

### 2.4. Study Selection and Data Extraction

Records were exported to Microsoft Excel (Microsoft Corporation, Redmond, WA, USA) and EndNote 21 (Clarivate, Philadelphia, PA, USA), and duplicates were removed through manual cross-checking. Two reviewers independently screened titles, abstracts, and full texts. Disagreements were resolved by consensus, with a third reviewer consulted when necessary. Extracted variables included study design, sample size, age, sex, training status, supplementation protocol, oral formulation (plant part, powder/extract/tablet form, extraction method, standardization or brand, and withanolide content when reported), comparator condition, intervention duration, exercise-performance outcomes, and data required for effect-size calculation.

Exercise-performance outcomes included time to exhaustion, peak or mean power output, maximal or peak oxygen uptake, maximal strength, handgrip strength, back-leg strength, bench-press or squat performance, repetition-based muscular endurance, vertical jump or countermovement jump height, standing long jump, medicine-ball throw distance, and other objectively measured performance indicators. When numerical data were not reported, corresponding authors were contacted by email. If no response was received, values were extracted from published figures using WebPlotDigitizer (Ankit Rohatgi, Pacifica, CA, USA) [26]. Extracted values were cross-checked by two reviewers to minimize transcription and figure-digitization errors.

### 2.5. Risk of Bias Assessment

Risk of bias was assessed using the Cochrane Risk of Bias 2 tool (RoB 2) [27]. The five standard domains were bias arising from the randomization process, bias due to deviations from intended interventions, bias due to missing outcome data, bias in measurement of the outcome, and bias in selection of the reported result. Because this review focused on exercise-performance testing, particular attention was given to placebo adequacy, allocation concealment, blinding implementation and credibility, assessor blinding, and standardization of performance-test procedures. Two reviewers independently assessed each study as low risk, some concerns, or high risk of bias; disagreements were resolved by consensus or third-reviewer adjudication.

### 2.6. Statistical Analysis

Only exercise-performance outcomes were included in the meta-analysis. For studies reporting pre–post changes, standardized mean differences were preferentially calculated from between-group differences in change scores. When change scores were unavailable, effect sizes were calculated from post-intervention means, standard deviations, and sample sizes. When baseline and post-intervention standard deviations were available but the standard deviation of change scores was not reported, the change-score standard deviation was estimated using a pre–post correlation coefficient of r = 0.60, with sensitivity analyses using r = 0.40 and r = 0.80. All effects were converted to Hedges’ g to correct for small-sample bias [28]. Positive values favored ashwagandha over placebo or control; outcomes for which lower values indicated better performance were reverse-coded before analysis.

Each effect size was identified by StudyID and EffectsizeID and included Hedges’ g, standard error, and sampling variance. Because trials commonly reported multiple performance outcomes from shared participants, a three-level random-effects model was used as the primary analysis to account for dependence among effect sizes [29]. This model decomposed total variance into sampling error, within-study between-effect-size variance, and between-study variance, with effect sizes nested within studies as random = ~1|StudyID/EffectsizeID. Models were estimated using restricted maximum likelihood (REML), with maximum likelihood (ML) used as a robustness check [30]. Pooled Hedges’ g, 95% confidence intervals, *p* values, 95% prediction intervals, and three-level I^2^ estimates were reported [31]. I^2^ values of 0–25%, 25–50%, 50–75%, and >75% were interpreted as low, moderate, substantial, and considerable heterogeneity, respectively [32].

Exercise-performance outcomes were classified according to their predominant energy system, neuromuscular mechanism, task duration, and adaptation profile. Cardiorespiratory endurance included VO_2_max, maximal aerobic power, time to exhaustion, and endurance tests. Muscular strength or muscular endurance included one-repetition maximum, handgrip strength, back-leg strength, isometric strength, and repetition-based tests. Power or explosive performance included jumping, throwing, peak power, mean power, and velocity-output measures. Categorical moderator analyses examined sex, training status, blinding design, exercise type, extract-dose category, intervention duration category, outcome domain, and oral formulation. Formulation was coded from trial reports by plant part and delivery form, including root powder or co-administered whole preparation, root extract, standardized root extract, aqueous root preparation, root-and-leaf extract, and extract tablet; brand names and standardization details were retained descriptively where available. Because formulation categories were sparse and frequently confounded with dosage, intervention duration, exercise modality, and training program, formulation analyses were considered exploratory and used to assess consistency rather than superiority of any product. The co-administered 12 g/day whole-preparation study was retained in the overall synthesis but was not interpreted as extract-equivalent dosing in extract-dose inferences. Continuous meta-regressions examined extract dose (mg/day) and intervention duration (weeks) using REML-based mixed-effects models; linear and quadratic models were fitted. Because several subgroups contained few independent studies or effect sizes, subgroup and meta-regression findings were interpreted according to effect direction, effect magnitude, confidence intervals, heterogeneity, and biological plausibility rather than *p* values alone.

To evaluate whether the handling of dependent effect sizes influenced the main estimate, two dependency-structure sensitivity analyses were conducted. The primary model retained all effect sizes within studies. A supplementary two-level model aggregated multiple effects from each study into a single study-level effect and re-estimated the overall effect under assumed within-study effect-size correlations of rho = 0.20, 0.40, and 0.80. This aggregation parameter was distinct from the pre–post correlation used to estimate change-score standard deviations. Publication bias and small-study effects were explored using contour-enhanced funnel plots and Egger’s regression test [33]. Because multiple correlated effect sizes were extracted from the same trials, these analyses were considered exploratory and interpreted alongside sensitivity analyses and GRADE ratings. Sensitivity analyses included REML versus ML estimation, leave-one-study-out analysis, leave-one-effect-size-out analysis, outlier and influence diagnostics, exclusion of low-precision effect sizes, restriction to double-blind trials, and repeated analyses under alternative correlation assumptions. All analyses were performed in R (R Foundation for Statistical Computing, Vienna, Austria) using the metafor package and related visualization packages [30].

### 2.7. Certainty of Evidence

The certainty of evidence for the main outcomes and key subgroup findings was assessed using GRADE [34]. Certainty was rated as high, moderate, low, or very low across risk of bias, inconsistency, indirectness, imprecision, and publication bias. Inconsistency was judged using I^2^, three-level variance decomposition, prediction intervals, and consistency of effect direction across outcome domains. Imprecision was assessed from confidence interval width, prediction intervals crossing the null, and the number of contributing studies and effects. Indirectness considered participant characteristics, formulation, dosage, intervention duration, and representativeness of performance outcomes. Studies with insufficient reporting of plant part, extraction method, degree of withanolide standardization, or active-constituent content were downgraded when this limited applicability or comparability.

## 3. Results

### 3.1. Study Selection

**Figure 1:**
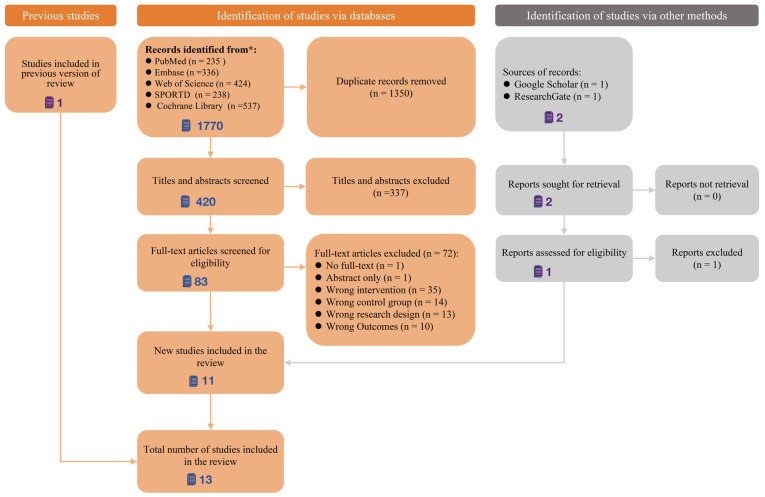
PRISMA 2020 flow diagram of study identification, screening, eligibility assessment, and inclusion. Database searching identified 1770 records; two additional records were identified through Google Scholar and ResearchGate, and one study was retained from a previous review. After duplicate removal (*n* = 1350), 420 records were screened, 337 were excluded at title/abstract screening, and 83 full-text articles were assessed. Seventy-two full-text articles were excluded for the eligibility reasons shown in the figure. The final synthesis included 13 randomized controlled trials contributing 79 exercise-performance effect sizes. The asterisk denotes records identified through the listed databases before duplicate removal.

The final search log identified 1770 database records, two additional records from other methods, and one study retained from previous reviews. After removal of 1350 duplicate records, 420 records were screened by title and abstract, 337 were excluded, and 83 full-text articles were assessed. Seventy-two full-text articles were excluded according to the reasons listed in Figure 1. Eleven newly identified studies from database searching, one study from previous reviews, and one study from other methods yielded 13 randomized controlled trials [35,36,37,38,39,40,41,42,43,44,45,46,47]. These trials compared oral ashwagandha supplementation with placebo or control conditions and contributed 79 exercise-performance effect sizes. The study selection process is summarized in Figure 1.

### 3.2. Study Characteristics

The 13 included randomized controlled trials provided data from 599 participants in the eligible comparisons included in the quantitative synthesis [35,36,37,38,39,40,41,42,43,44,45,46,47]. This number refers to participants contributing to the extracted intervention–control comparisons and may differ from the total randomized or enrolled sample reported in individual trials, because non-eligible arms from multi-arm studies and participants not included in analyzable comparisons were excluded where applicable. The full trial sample sizes reported across studies ranged from 30 to 108 participants. Participants were mostly healthy or physically active young adults and athletes, with one late-adolescent athlete cohort (17.4 ± 1.7 years) and one study reporting an explicit 18–40-year age range; age reporting was otherwise heterogeneous across trials. At the trial level, four studies were male-only, one was female-only, three clearly included both sexes, and five did not provide complete sex-specific counts in the information extracted for this review. All interventions were orally administered, but product forms differed materially, including standardized root extracts such as KSM-66, nonbranded root extracts, aqueous root preparations or capsules, a Sensoril root-and-leaf aqueous extract, extract tablets, and one co-administered *Withania somnifera* whole-preparation protocol with milk. Extract-based dosages were 500, 600, or 1000 mg/day, whereas the co-administered whole-preparation study used 12 g/day and was not considered extract-equivalent for dose interpretation. Intervention durations ranged from 28 days to 12 weeks. Across studies, formulation was closely linked to dose, duration, training exposure, and outcome domain; therefore, the available evidence did not permit a reliable inference that one oral form produced superior performance effects. Study characteristics are summarized in Table 1.

### 3.3. Overall Exercise Performance

**Figure 2:**
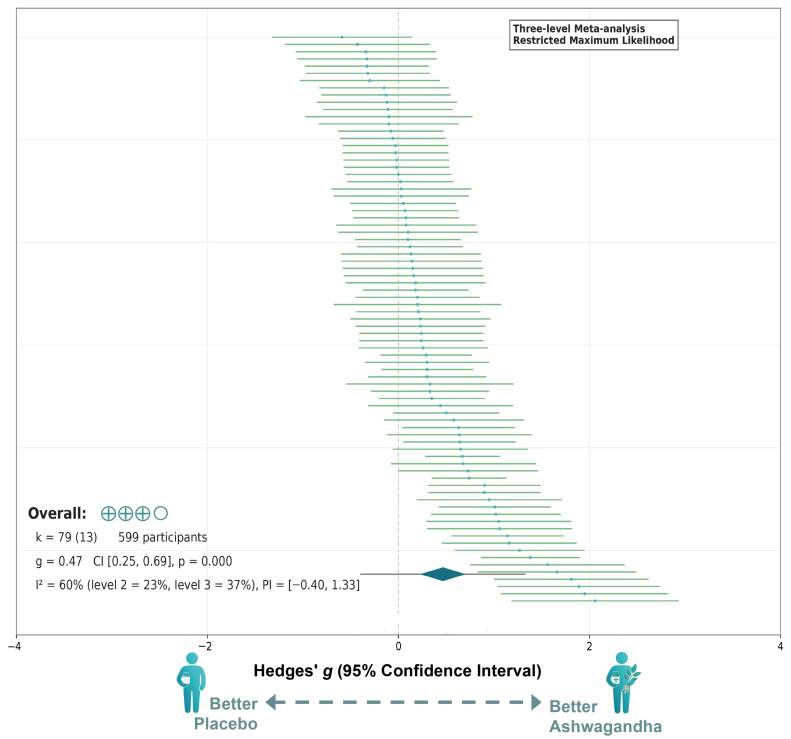
Main pooled effect of oral ashwagandha on overall exercise performance. Model: k = 79 effects from 13 studies and 599 participants; Hedges’ g = 0.47, 95% CI [0.25, 0.69], *p* < 0.001; I^2^ = 60%; 95% prediction interval [−0.40, 1.33]. The vertical dashed line denotes the null effect; the diamond denotes the pooled estimate; the horizontal line denotes the 95% prediction interval; and icons indicate the direction of better performance.

The three-level random-effects model showed that oral ashwagandha supplementation was associated with improved overall exercise performance (Hedges’ g = 0.47, 95% CI [0.25, 0.69], *p* < 0.001; I^2^ = 60%; 95% prediction interval [−0.40, 1.33]; moderate certainty) (Figure 2). This estimate corresponds to a small-to-moderate positive effect. However, the prediction interval crossed the null, indicating that the true effect in future comparable trials may range from no benefit or a slight negative effect to a more pronounced positive effect. Ashwagandha should therefore not be interpreted as producing consistent ergogenic benefits across all performance contexts; rather, its effects appear to depend on outcome domain, participant characteristics, study design, and supplementation protocol.

Variance decomposition showed that within-study variance accounted for 23% of total variance at level 2, between-study variance accounted for 37% at level 3, and the remaining variance reflected sampling error. Under the criterion of Hunter and Schmidt [32], a sampling-error proportion below 75% indicates meaningful heterogeneity. Thus, although the overall pooled estimate is useful as a summary, it does not identify the specific performance capacities in which ashwagandha is most likely to act. Moderator analyses were therefore central to interpretation.

### 3.4. Moderator Analyses

By study design, the positive effect remained significant in double-blind randomized trials (g = 0.46, 95% CI [0.20, 0.73], *p* < 0.001; moderate certainty) and was also observed in single-blind trials (g = 0.52, 95% CI [0.04, 1.01], *p* = 0.039; low certainty). The between-design difference was not statistically significant (P_between = 0.910), suggesting that the overall effect was not explained solely by weaker blinding.

Sex-based subgroup analyses showed the clearest effect in mixed-sex samples (g = 0.56, 95% CI [0.33, 0.78], *p* < 0.001; low certainty). The estimate in female samples was positive but imprecise (g = 0.52, 95% CI [−0.20, 1.25], *p* = 0.149; low certainty), and the estimate in male samples was smaller and non-significant (g = 0.20, 95% CI [−0.10, 0.49], *p* = 0.183; low certainty). The between-sex test was not significant (P_between = 0.672). These results do not establish a sex-specific response, although the limited number of sex-specific studies reduces confidence.

By training status, trained individuals showed a significant positive effect (g = 0.65, 95% CI [0.27, 1.03], *p* = 0.002; moderate certainty), and untrained or non-systematically trained individuals also showed a smaller significant effect (g = 0.33, 95% CI [0.07, 0.59], *p* = 0.015; moderate certainty). The between-group difference was not significant (P_between = 0.156). Thus, the available evidence does not restrict potential benefits to trained individuals, although the larger point estimate in trained samples is biologically plausible because higher training loads may increase the relevance of recovery and stress-regulation pathways.

Exercise type was the clearest moderator (P_between = 0.006). Aerobic endurance outcomes showed the most stable benefit (g = 0.54, 95% CI [0.22, 0.85], *p* = 0.002; moderate certainty). This pattern suggests that ashwagandha may preferentially influence performance dimensions related to cardiorespiratory oxygen delivery, peripheral oxygen utilization, fatigue tolerance, or sustained exercise capacity. Strength outcomes showed a positive point estimate but did not reach conventional statistical significance (g = 0.48, 95% CI [−0.04, 1.01], *p* = 0.068; moderate certainty). Power performance showed no clear improvement (g = 0.18, 95% CI [−0.18, 0.54], *p* = 0.982; low certainty), and muscular endurance remained imprecise (g = 0.29, 95% CI [−0.83, 1.41], *p* = 0.303; very low certainty).

**Figure 3:**
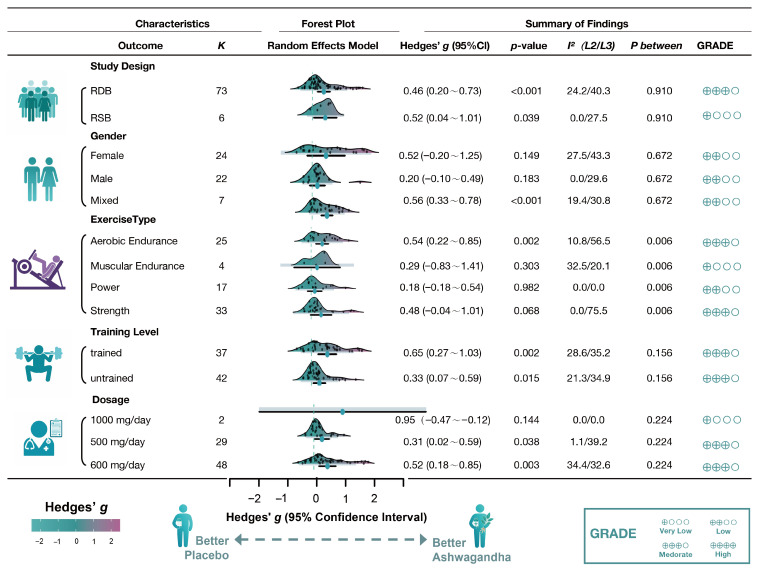
Moderator analyses of the effects of oral ashwagandha supplementation on exercise performance. Subgroup results from three-level random-effects models are shown according to study design, sex, exercise type, training status, and extract-dose category. Forest or density plots show pooled effect-size distributions for each subgroup, with Hedges’ g, 95% CI, *p* value, level-2 and level-3 I^2^ estimates, between-subgroup tests, and GRADE certainty ratings. Dose-specific findings should be interpreted cautiously because extract standardization differed across trials and the 1000 mg/day subgroup contained very few effect sizes. Exercise type was the clearest source of heterogeneity; evidence was most stable for aerobic endurance, strength showed a possible positive effect, and evidence for power and muscular endurance remained limited.

Dosage analyses suggested a possible dose-related pattern, but the evidence was not sufficient to establish a causal dose–response relationship. The 500 mg/day subgroup showed a small but significant effect (g = 0.31, 95% CI [0.02, 0.59], *p* = 0.038; moderate certainty), whereas the 600 mg/day subgroup showed a larger and more stable effect (g = 0.52, 95% CI [0.18, 0.85], *p* = 0.003; moderate certainty). The 1000 mg/day subgroup had the largest point estimate (g = 0.95), but it included very few effect sizes and was not statistically significant (*p* = 0.144; very low certainty). The between-dose test was not significant (P_between = 0.224). Therefore, these findings are better interpreted as a possible dose signal than as definitive evidence of dose dependence. At present, 500–600 mg/day appears to be the most evidence-supported and stable range (Figure 3).

Exploratory formulation-level analyses were descriptive because oral forms were unevenly represented. Positive endurance effects were observed primarily in studies using root-based preparations, including standardized root extracts and aqueous root capsules, whereas strength-related outcomes were mostly contributed by root-extract studies with concurrent resistance training or by a root-and-leaf extract trial. However, the formulation category was confounded with dose, study design, training intervention, and performance domain. No oral form, extract brand, or plant-part category could therefore be identified as independently superior.

### 3.5. Dose and Duration Meta-Regression

**Figure 4:**
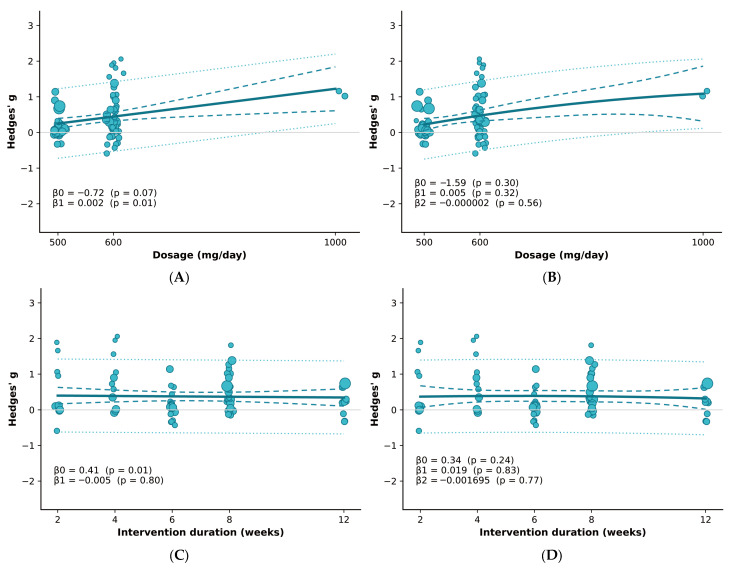
Meta-regression analyses of ashwagandha dosage and intervention duration. Panels (**A**,**B**) show the linear and nonlinear meta-regression models for dosage. Panels (**C**,**D**) show the linear and nonlinear models for intervention duration. Solid lines represent fitted effects; dashed lines represent confidence intervals; dotted lines represent prediction or uncertainty bounds; grey horizontal lines denote the null effect; and circle size reflects effect-size precision or relative weighting. The dose model indicated an exploratory positive linear association, whereas intervention duration showed no clear linear or nonlinear association with exercise-performance effect size.

The continuous extract-dose meta-regression showed a statistically significant positive association between daily ashwagandha extract dose and exercise-performance effect size (β0 = −0.72, *p* = 0.070; β1 = 0.002, *p* = 0.010) (Figure 4A), but this association should not be interpreted as proof that higher doses are more effective. Expressed per 100 mg/day, the slope corresponded to an approximate increase of 0.20 in Hedges’ g. The quadratic dose model did not show a significant nonlinear relationship (β0 = −1.59, *p* = 0.300; β1 = 0.005, *p* = 0.320; β2 = −0.000002, *p* = 0.560) (Figure 4B). These results are more consistent with an approximately linear exploratory dose signal than with a clear curvilinear or plateau-shaped pattern. However, because most data were concentrated around 500 and 600 mg/day, the 1000 mg/day evidence was extremely sparse, and one whole-preparation gram-dose study was not extract-equivalent; this analysis should be considered hypothesis-generating and cannot support recommending higher doses.

Intervention duration was not significantly associated with effect size in the linear model (β0 = 0.41, *p* = 0.010; β1 = −0.005, *p* = 0.800) (Figure 4C), and the quadratic duration model also showed no significant nonlinear time-response relationship (β0 = 0.34, *p* = 0.240; β1 = 0.019, *p* = 0.830; β2 = −0.001695, *p* = 0.770) (Figure 4D). Duration may still matter biologically, but the available evidence suggests that it cannot be interpreted independently from training stimulus, baseline stress, sleep, recovery conditions, and formulation characteristics.

### 3.6. Risk of Bias, Publication Bias, and Certainty of Evidence

**Figure 5:**
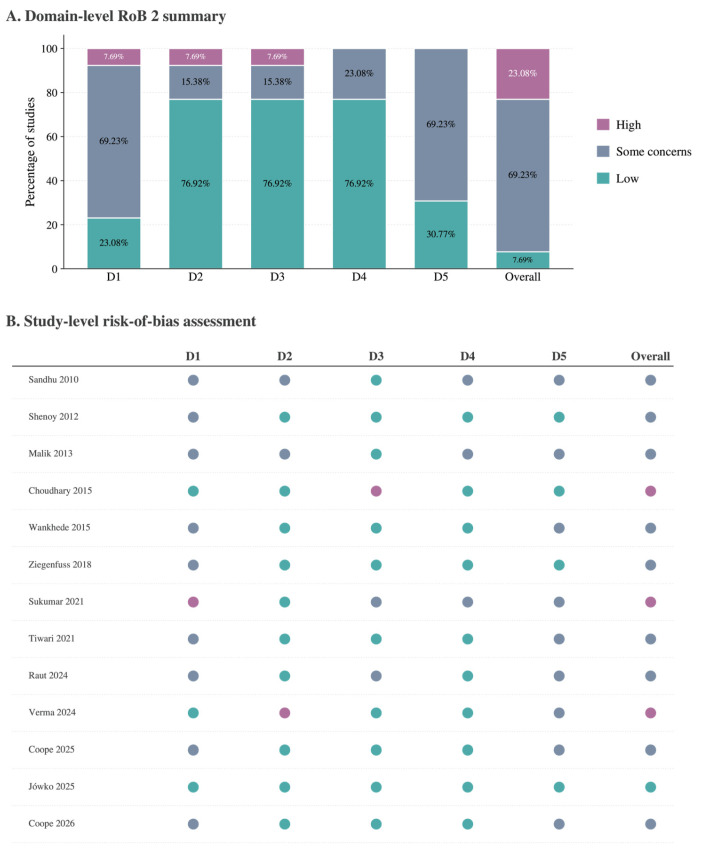
Risk of bias assessment using the Cochrane RoB 2 tool. Panel (**A**) summarizes domain-level judgments. Panel (**B**) shows study-level judgments for D1 (randomization process), D2 (deviations from intended interventions), D3 (missing outcome data), D4 (outcome measurement), D5 (selection of the reported result), and overall risk of bias [35,36,37,38,39,40,41,42,43,44,45,46,47].

RoB 2 assessments are presented in Figure 5. Of the 13 trials, one was rated as low risk of bias overall, nine as having some concerns, and three as high risk. The randomization domain showed the greatest uncertainty: 23.08% of studies were rated low risk, 69.23% some concerns, and 7.69% high risk. Deviations from intended interventions and missing outcome data were rated low risk in 76.92% of studies, some concerns in 15.38%, and high risk in 7.69%. Outcome measurement was rated low risk in 76.92% and some concerns in 23.08%. Selection of reported results was rated low risk in 30.77% and some concerns in 69.23%. These results indicate that, despite the use of randomized and placebo-controlled designs, the evidence is constrained by incomplete reporting of randomization and allocation concealment, limited verification of blinding credibility, and possible selective reporting. Consequently, the statistically significant pooled effect should be interpreted as provisional rather than definitive, and conclusions should remain aligned with the moderate-to-low certainty of much of the evidence.

Small-study effects and potential publication bias were assessed using contour-enhanced funnel plots, Egger’s regression, and the trim-and-fill procedure within a conventional two-level random-effects meta-analytic framework. The contour-enhanced funnel plot showed no clear pattern of asymmetry suggestive of one-sided missing studies. Egger’s regression provided no evidence of statistically significant small-study effects, with an intercept of 0.82 (SE = 0.98, *p* = 0.406). In the subsequent trim-and-fill analysis, the default side-selection procedure indicated the left side, but no missing effect sizes were imputed. The adjusted pooled estimate was essentially unchanged from the unadjusted estimate (g = 0.47, 95% CI [0.25, 0.69]; Supplementary File S3), suggesting that the primary effect was relatively robust to potential publication bias.

These diagnostics should nevertheless be interpreted cautiously. Multiple correlated effect sizes were derived from the same set of trials, and several subgroup analyses included only a small number of independent studies. Accordingly, the available evidence cannot fully rule out small-study effects or publication bias, particularly in subgroups with limited independent evidence. Replicability diagnostics [48] further indicated low median observed statistical power (14.0%), a success rate of 26.6%, and a low R-index of 1.4% (Supplementary File S4), suggesting that the evidence base remains constrained by small samples and low-powered studies. These findings warrant cautious interpretation of the pooled results.

The certainty-of-evidence assessment indicated moderate certainty for the overall effect (Supplementary File S5), suggesting that oral *Withania somnifera* supplementation may improve exercise performance on average, but that further high-quality trials could change the magnitude and contextual interpretation of this estimate. This inference remains limited by the small number of independent studies, the non-independence of correlated effect sizes, and imprecision in several outcomes. Among the outcome domains, aerobic endurance showed the most stable evidence, with a consistent direction of effect, confidence intervals that did not cross the line of no effect, and a comparatively larger number of independent studies; this domain was therefore rated as moderate certainty. Strength outcomes also showed a positive trend, but wider confidence intervals and between-study differences require cautious interpretation. By contrast, the evidence for power and muscular endurance outcomes was weaker, primarily because of the small number of effect sizes, limited independent studies, imprecise estimates, and lower certainty of evidence. These outcomes therefore do not yet support definitive conclusions.

### 3.7. Sensitivity Analyses

**Figure 6:**
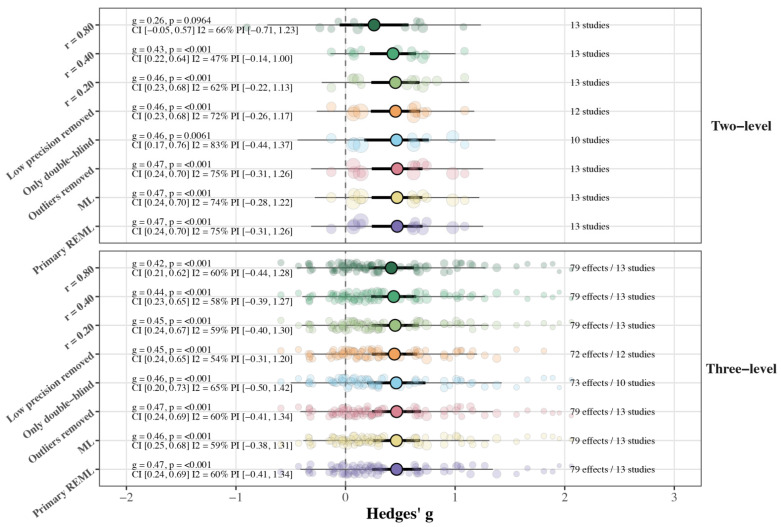
Sensitivity analyses for the primary pooled effect. Sensitivity analyses included REML versus ML estimation, exclusion of low-precision effect sizes, restriction to double-blind trials, outlier removal, and alternative within-study correlation assumptions in two-level and three-level models. Across three-level sensitivity analyses, effect sizes remained positive and statistically significant, supporting the robustness of the main pooled effect while retaining substantial heterogeneity.

Sensitivity analyses generally supported the robustness of the main finding (Figure 6). The primary three-level REML model retained all 79 effect sizes nested within 13 studies and showed a significant positive effect (g = 0.47, 95% CI [0.24, 0.69], *p* < 0.001). Under ML estimation, outlier exclusion, restriction to double-blind trials, exclusion of low-precision effect sizes, and alternative within-study correlation assumptions, three-level estimates remained between g = 0.42 and g = 0.47 and all remained statistically significant (Supplementary File S6).

In supplementary two-level aggregated models, multiple effects from each study were collapsed into a single study-level effect. The effect remained significant under most assumptions but became non-significant under the most conservative high-correlation scenario (rho = 0.80). This pattern indicates that aggregation can reduce precision when dependence is handled conservatively. Because the three-level model better reflects the nested data structure and remained significant across all sensitivity conditions, the primary conclusion supports a stable overall positive effect, while acknowledging substantial heterogeneity and uncertainty in future study effects.

## 4. Discussion

This systematic review and three-level meta-analysis found that oral ashwagandha supplementation was associated with a small-to-moderate average improvement in overall exercise performance. Importantly, the 95% prediction interval crossed zero, indicating that future comparable trials may observe no benefit, or even a slight negative effect, under some conditions. The effect was most consistent for aerobic endurance, showed a possible but less certain benefit for strength, and remained weak or unstable for power and muscular endurance. These findings indicate that ashwagandha should not be characterized as a broad-spectrum ergogenic aid for all forms of performance. Rather, its effects appear to be domain-specific and dependent on the physiological demands of the task, training context, dosage, and product characteristics.

Several included trials and related reviews reported improvements in VO_2_max or time-to-exhaustion outcomes after ashwagandha supplementation [35,36,37,38,39,40,41,42,43,49,50,51,52]. VO_2_max and related endurance outcomes reflect integrated oxygen uptake, transport, and utilization and are constrained by cardiovascular delivery, blood oxygen-carrying capacity, mitochondrial oxidative phosphorylation, metabolic efficiency, and fatigue regulation [53,54,55,56,57]. The present findings suggest that the benefit may arise not from a single pathway but from the convergence of peripheral oxygen utilization, energy-metabolism support, stress regulation, and fatigue tolerance.

One plausible pathway involves oxygen transport and peripheral oxygen utilization. Some ashwagandha trials have reported changes in hemoglobin or red-blood-cell-related outcomes [37,41,58,59]. Improved oxygen transport could delay the shift toward greater anaerobic contribution during prolonged exercise, whereas better mitochondrial function or lower oxidative stress could support ATP resynthesis during sustained effort [56,57]. These mechanisms remain inferential because most trials did not directly measure mitochondrial function, muscle oxygenation, or oxygen-transport biomarkers alongside performance outcomes. Future trials should pair performance tests with mechanistic markers to identify whether endurance effects are mediated by oxygen delivery, peripheral oxygen extraction, or central fatigue.

Stress regulation may also contribute to endurance responses. Prolonged or high-load training increases the importance of sleep, psychological readiness, perceived fatigue, and HPA-axis regulation. Ashwagandha has been reported to reduce perceived stress and cortisol in several clinical contexts [17,18,19,60,61]. Lower chronic stress load may improve recovery, metabolic regulation, and test-day readiness, thereby allowing participants to sustain effort during endurance tasks. This interpretation is consistent with the larger point estimate in trained individuals, although the training-status moderator was not statistically significant.

Strength outcomes showed a positive but less stable effect. Strength adaptations depend on neural drive, motor-unit recruitment, muscle cross-sectional area, protein synthesis, endocrine status, and recovery after resistance training [62,63,64,65,66]. Ashwagandha is unlikely to act as an acute strength enhancer in the manner of caffeine. Instead, it may support resistance-training adaptation by improving recovery conditions, modulating stress-related catabolism, and limiting excessive inflammation or oxidative damage. Prior trials and reviews suggest possible influences on testosterone, DHEA, cortisol, muscle recovery, and muscle-cell differentiation [17,52,65,66]. These mechanisms are biologically plausible, but the current performance evidence is not sufficiently consistent to make strong strength-specific claims.

The limited effects for power and muscular endurance are consistent with the proposed mechanism. Power and explosive performance depend heavily on rapid neural excitation, fast motor-unit recruitment, type II fiber contribution, and phosphagen system availability [67,68]. Supplements with strong effects on these outcomes often act through central stimulation, phosphocreatine availability, or buffering capacity [5,6,8,67,68]. Ashwagandha’s primary profile—chronic stress modulation, sleep and recovery support, and redox regulation—is more indirect for short-duration explosive tasks. Muscular endurance findings were also imprecise, probably because tests differed substantially across studies. Repetition-to-failure tasks, sustained isometric contractions, and whole-body conditioning tests share the label of muscular endurance but are limited by different combinations of local metabolism, pain tolerance, pacing, and motor control.

Oxidative stress and inflammation pathways provide another coherent explanation. Exercise increases reactive oxygen species production [69,70]. Appropriate redox signaling is necessary for adaptation, but excessive oxidative stress can damage lipids, proteins, and mitochondrial structures, reduce force production, and slow recovery [69,70,71]. Ashwagandha should therefore not be framed simply as a free-radical scavenger; a more plausible interpretation is modulation of redox balance. Withaferin A has been shown to activate Nrf2-related antioxidant pathways and upregulate antioxidant proteins such as HO-1 [72]. Withanolides may also influence NF-kappaB signaling and inflammatory responses [73]. For athletes, such effects could support recovery after high-intensity or high-volume training, although excessive antioxidant supplementation can theoretically blunt some training adaptations [71]. This trade-off should be addressed directly in future work.

Sleep and GABAergic pathways may be particularly relevant to the sports context. Sleep influences glycogen restoration, neuromuscular recovery, hormonal rhythmicity, immune function, and psychological readiness [74,75]. Meta-analytic and clinical evidence suggests that ashwagandha can improve sleep quality, sleep latency, total sleep time, and sleep efficiency [20,21,76]. Mechanistic evidence indicates possible GABAergic activity, including interactions with GABAA and related receptors [77]. If ashwagandha improves sleep or reduces stress during a training cycle, performance benefits may emerge indirectly through better recovery and training consistency rather than immediate pre-exercise stimulation.

The dosage findings should be interpreted cautiously. The 500–600 mg/day range was the most evidence-supported and stable in the present analysis, whereas the apparently larger estimate at 1000 mg/day was based on sparse evidence. Comparing milligram doses across trials is also imperfect because ashwagandha products differ in plant part, extraction ratio, withanolide content, withaferin A content, and quality-control procedures [13,15,16,50,51]. Products marketed as root extract, aqueous extract, KSM-66, Sensoril, Shoden, or other preparations may not provide equivalent active-constituent exposure. Future trials should report the extraction method, plant part, withanolide standardization, analytical verification, batch information, and third-party testing.

Safety and practical implementation require careful framing. The included trials generally reported acceptable short-to-medium-term tolerability at commonly used doses [35,36,37,38,39,40,41,42,43,44,45,46,47,52]. Mild adverse effects may include somnolence, dizziness, or gastrointestinal discomfort. Nevertheless, case reports have linked ashwagandha or ashwagandha-containing products with reversible liver injury [78]. These reports do not prove common hepatotoxicity at standard doses, but they reinforce the need for caution regarding product source, dose, duration, concurrent supplement use, and individual liver-health status. Competitive athletes should use third-party-tested products to reduce the risk of contamination with undeclared or prohibited substances [2].

This review has several strengths. It used a three-level meta-analytic model to retain multiple outcomes while accounting for dependence among effect sizes [29]. It also separated exercise-performance constructs, enabling clearer interpretation than a single undifferentiated pooled estimate. The review integrated risk of bias, prediction intervals, sensitivity analyses, and GRADE certainty, which provides a more conservative interpretation of a heterogeneous evidence base.

Several limitations should be emphasized. The evidence base was small and uneven, with only 13 independent trials and many subgroup analyses including few studies or effect sizes. Trials differed in formulation, dosage, intervention duration, participant training status, concurrent training stimulus, and performance tests. Many studies incompletely reported training load, diet, sleep, stress, supplement adherence, blinding credibility, and product standardization. The English-language restriction may have introduced language bias. Publication-bias analyses were exploratory because of dependent effect sizes and a limited number of independent studies. Finally, several mechanistic interpretations remain indirect because most trials did not measure relevant biomarkers alongside performance outcomes. These limitations reduce confidence in dose-, sex-, training status-, and formulation-specific inferences and prevent conclusions about the superiority of any oral form or extract brand.

Future research should use preregistered, adequately powered, double-blind, placebo-controlled designs, preferably in trained participants and athletes [24,25,27,34]. Trials should prespecify primary performance outcomes and statistical handling of multiple outcomes, apply concealed allocation and blinding-credibility checks, and standardize or carefully report training programs, diet, sleep, psychological stress, adherence, and adverse events. Product characterization should include plant part, extraction method, extraction ratio, withanolide and withaferin A content, batch-level analytical verification, and third-party testing. Mechanistic panels should include hemoglobin, red-blood-cell indices, ferritin where relevant, cortisol, testosterone, DHEA, inflammatory markers, oxidative stress markers, muscle damage indices, sleep metrics, and product-specific withanolide profiles. Sex-specific designs should report menstrual-cycle phase, hormonal contraceptive use, iron status, energy availability, sleep, and stress to determine whether responses differ between men and women.

From a practical perspective, ashwagandha is best positioned as a chronic recovery- and adaptation-support supplement rather than as an acute pre-event stimulant. For healthy adults, standardized 500–600 mg/day preparations may be reasonable only as a conditional, context-specific option rather than a general ergogenic recommendation. Use should consider product standardization, training load, sleep status, stress burden, medical history, liver-health status, concurrent supplement or medication use, and anti-doping quality control. Claims for explosive power or muscular endurance enhancement should remain conservative until more domain-specific trials are available.

## 5. Conclusions

Oral ashwagandha supplementation may produce small-to-moderate average improvements in exercise performance, with the most consistent evidence for aerobic endurance and a possible but less certain effect on strength-related outcomes. However, the prediction interval crossing zero, substantial heterogeneity, sparse subgroup evidence, product-standardization differences, and risk of bias concerns mean that the findings should be interpreted cautiously. The benefits are more plausibly explained by chronic modulation of recovery, stress regulation, sleep, redox balance, inflammation, oxygen transport, and training adaptation than by acute stimulation or a single metabolic pathway. Evidence for power and muscular endurance remains limited. Current evidence supports only conditional use of standardized 500–600 mg/day preparations in healthy adults when product quality, training context, medical history, and anti-doping safeguards are considered. Future trials should be larger, preregistered, double-blind, formulation-standardized, and adequately powered; they should include sex-stratified analyses, rigorous blinding checks, harmonized performance tests, mechanistic biomarkers, and prospective adverse-event monitoring.

## Figures and Tables

**Table 1:** Characteristics of the included randomized controlled trials.

Study	Design	Participants	Oral Ashwagandha Form and Dose	Control and Duration	Exercise-Performance Outcomes
Sandhu et al. (2010) [35]	RCT	Healthy young adults; total *n* = 40; four-arm trial	Standardized *Withania somnifera* root extract; 500 mg/day	Placebo; 8 weeks	Maximal velocity (m/s); average absolute power (kW/m^2^); VO_2_max (mL/kg/min)
Shenoy et al. (2012) [36]	RCT	Elite Indian cyclists; total *n* = 40; 20 men and 20 women	Aqueous *Withania somnifera* root capsules; 500 mg twice daily	Starch placebo; 8 weeks	GXT time to exhaustion (min); VO_2_max (mL/kg/min)
Malik et al. (2013) [37]	RCT	Male field hockey players; total *n* = 32; mean age 17.4 ± 1.7 years	Aqueous *Withania somnifera* root capsules; 500 mg twice daily	Starch placebo; 8 weeks	VO_2_max (mL/kg/min)
Choudhary et al. (2015) [38]	DB-RCT	Healthy exercising adults; total *n* = 50	KSM-66 *Withania somnifera* root extract; 300 mg twice daily	Sucrose placebo; 12 weeks	VO_2_max (mL/kg/min)
Wankhede et al. (2015) [39]	DB-RCT	Healthy young men with limited resistance-training experience; total *n* = 57	*Withania somnifera* root extract; 300 mg twice daily	Starch placebo; 8 weeks; both groups completed resistance training	Bench press 1RM (kg); leg extension 1RM (kg)
Ziegenfuss et al. (2018) [40]	DB-RCT	Recreationally active healthy men; total *n* = 40	Sensoril *Withania somnifera* root-and-leaf aqueous extract; 500 mg/day	Placebo; 12 weeks	7.5 km time trial (s); peak power across all squat sets (W); squat 1RM; squat repetitions across all sets (reps); bench press 1RM (kg); peak power across all bench-press sets (W); bench-press repetitions across all sets (reps)
Sukumar et al. (2021) [41]	RCT	Healthy adults; total *n* = 108; age range reported as 18–40 years	*Withania somnifera* co-administered with milk; 12 g/day	Milk-only control; 60 days	VO_2_max (mL/kg/min)
Tiwari et al. (2021) [42]	DB-RCT	Healthy exercising adults; total *n* = 50; 25 per group	*Withania somnifera* root extract; 300 mg twice daily	Placebo; 8 weeks	VO_2_max (mL/kg/min)
Verma et al. (2024) [43]	DB-RCT	Physically active men and women; total *n* = 80; 40 per group	Standardized *Withania somnifera* root extract; 300 mg twice daily	Placebo; 8 weeks; both groups completed resistance training	Bench press 1RM (kg); leg press 1RM (kg); VO_2_max (mL/kg/min)
Raut et al. (2024) [44]	DB-RCT	Healthy volunteers; *n* = 62 enrolled; approximately *n* = 51 analyzed	*Withania somnifera* extract tablets; 250 mg twice daily	Placebo; 60 days	Six-minute cycle-ergometer distance (km); six-minute cycle-ergometer speed (km/h); back-leg-press dynamometry (kg); handgrip strength (kg)
Coope et al. (2025) [45]	RCT	Female soccer players; total *n* = 30; 15 per group	*Withania somnifera* root extract; 600 mg/day	Placebo; 28 days	Handgrip strength (kg); left-handgrip strength (kg); right-handgrip strength (kg); medicine-ball throw (cm); CMJ (cm); peak power (W)
Jówko et al. (2025) [46]	DB-RCT	Healthy non-athletic men; total *n* = 33; 17 intervention and 16 control	Ashwagandha preparation; formulation/standardization not clearly reported; 600 mg/day	Placebo; 8 weeks; both groups completed rowing-ergometer HIIT three times per week	Maximal aerobic power (W); relative maximal aerobic power (W/kg); VO_2_max (mL/kg/min)
Coope et al. (2026) [47]	DB-RCT	Team-sport athletes from rugby, water polo, and soccer; total *n* = 56; 28 per group; men and women included	KSM-66 *Withania somnifera* root extract; 600 mg/day	Chickpea-flour placebo; 42 days	Squat 1RM; bench press 1RM (kg); deadlift 1RM (kg); Bronco test time (s); clean 1RM (kg); broad jump (cm); CMJ (cm); handgrip strength (kg); pull-ups (reps)

Note. RCT, randomized controlled trial; DB-RCT, double-blind randomized controlled trial; VO_2_max, maximal oxygen uptake; 1RM, one-repetition maximum; CMJ, countermovement jump; HIIT, high-intensity interval training. For multi-arm studies, only the ashwagandha-only intervention group and the corresponding placebo or control group were extracted for the primary synthesis; combined-intervention arms were excluded. The oral form and dose are reported as described by the original trial authors. Whole-preparation gram dosing should not be assumed to be equivalent to extract milligram dosing. Where extract standardization, withanolide content, or brand information was not reported, no equivalence between products should be assumed.

## Data Availability

The original contributions presented in this study are included in the article/Supplementary Materials. Further inquiries can be directed to the corresponding authors.

## Supplementary Materials

The following supporting information can be downloaded at: https://www.mdpi.com/article/10.3390/nu18121915/s1, File S1: PRISMA 2020 checklist; File S2: Database-specific search strategies; File S3: Small-study effects, Egger regression, trim-and-fill diagnostics, and moderator funnel plots; File S4: Power visualization, replicability diagnostics, and moderator power plots; File S5: GRADE certainty-of-evidence assessment; File S6: Leave-one-out sensitivity analyses at effect-size and study levels.
