# Supplementary material for: Effects of Withania somnifera (Ashwagandha) Supplementation on Exercise Performance: A Systematic Review and Three-Level Meta-Analysis
Source: Nutrients. 2026 Jun 12;18(12):1915. doi: 10.3390/nu18121915 (PMC13304793; doi:10.3390/nu18121915)

Electronic Supplementary Material

Effects of Withania somnifera (Ashwagandha) Supplementation on Exercise Performance: A Systematic Review and Three-Level Meta-Analysis

This supplementary file is structured to match the main manuscript and contains the PRISMA 2020 checklist, database-specific search strategies, small-study-effect diagnostics, power and replicability diagnostics, GRADE certainty assessment, and leave-one-out sensitivity analyses.

Electronic Supplementary Material Contents

Number	Material	Page
1	Electronic Supplementary Material File S1 (PRISMA 2020 checklist)	P2-P3
2	Electronic Supplementary Material File S2 (Database-specific search strategies)	P4
3	Electronic Supplementary Material File S3 (Small-study effects, Egger regression, trim-and-fill diagnostics, and moderator funnel plots)	P5-P6
4	Electronic Supplementary Material File S4 (Power visualization, replicability diagnostics, and moderator power plots)	P7-P8
5	Electronic Supplementary Material File S5 (GRADE certainty-of-evidence assessment)	P9
6	Electronic Supplementary Material File S6 (Leave-one-out sensitivity analyses at effect-size and study levels)	P10-P12

Electronic Supplementary Material File S1

PRISMA 2020 checklist

The checklist below maps PRISMA 2020 items to the corresponding sections of the manuscript. Page numbers should be updated after journal typesetting if the manuscript pagination changes.

Section and topic	Item #	Checklist item	Location
TITLE	1	Identify the report as a systematic review.	Title page
ABSTRACT	2	See the PRISMA 2020 for Abstracts checklist.	Abstract
INTRODUCTION: Rationale	3	Describe the rationale for the review in the context of existing knowledge.	Introduction
INTRODUCTION: Objectives	4	Provide an explicit statement of the objective(s) or question(s) the review addresses.	Introduction, final paragraph
METHODS: Eligibility criteria	5	Specify the inclusion and exclusion criteria for the review and how studies were grouped for the syntheses.	Materials and Methods: Eligibility criteria
METHODS: Information sources	6	Specify all databases, registers, websites, organisations, reference lists and other sources searched or consulted, and the date of last search.	Materials and Methods: Information sources and search strategy; File S2
METHODS: Search strategy	7	Present the full search strategies for all databases, registers and websites, including any filters and limits used.	File S2
METHODS: Selection process	8	Specify methods used to decide whether a study met inclusion criteria, including number of reviewers and independence.	Materials and Methods: Study selection and data extraction
METHODS: Data collection process	9	Specify methods used to collect data from reports, including number of reviewers, independence, and author contact or figure extraction procedures.	Materials and Methods: Study selection and data extraction
METHODS: Data items	10a	List and define all outcomes for which data were sought.	Materials and Methods: Study selection and data extraction; Statistical analysis
METHODS: Data items	10b	List and define other variables for which data were sought and describe assumptions for missing or unclear information.	Materials and Methods: Study selection and data extraction; Statistical analysis
METHODS: Study risk of bias assessment	11	Specify methods used to assess risk of bias in the included studies.	Materials and Methods: Risk of bias assessment
METHODS: Effect measures	12	Specify effect measures used in the synthesis or presentation of results.	Materials and Methods: Statistical analysis
METHODS: Synthesis methods	13a	Describe processes used to decide which studies were eligible for each synthesis.	Materials and Methods: Eligibility criteria; Statistical analysis
METHODS: Synthesis methods	13b	Describe data preparation for presentation or synthesis, including conversions and handling of missing summary statistics.	Materials and Methods: Statistical analysis
METHODS: Synthesis methods	13c	Describe methods used to tabulate or visually display results of individual studies and syntheses.	Materials and Methods: Statistical analysis; Figures 2-6
METHODS: Synthesis methods	13d	Describe methods used to synthesize results and provide a rationale for the model(s).	Materials and Methods: Statistical analysis
METHODS: Synthesis methods	13e	Describe methods used to explore possible causes of heterogeneity.	Materials and Methods: Statistical analysis; Moderator analyses
METHODS: Synthesis methods	13f	Describe sensitivity analyses conducted to assess robustness.	Materials and Methods: Statistical analysis; Results: Sensitivity analyses; File S6
METHODS: Reporting bias assessment	14	Describe methods used to assess risk of bias due to missing results.	Materials and Methods: Statistical analysis; Results: Risk of bias, publication bias, and certainty of evidence; File S3
METHODS: Certainty assessment	15	Describe methods used to assess certainty in the body of evidence.	Materials and Methods: Certainty of evidence; File S5
RESULTS: Study selection	16a	Describe results of the search and selection process, ideally using a flow diagram.	Results: Study selection; Figure 1
RESULTS: Study selection	16b	Cite studies that might appear to meet inclusion criteria but were excluded, and explain why.	Figure 1 and screening records
RESULTS: Study characteristics	17	Cite each included study and present its characteristics.	Results: Study characteristics; Table 1
RESULTS: Risk of bias in	18	Present assessments of risk of bias for each included study.	Results: Risk of bias; Figure 5

Section and topic	Item #	Checklist item	Location
studies			
RESULTS: Results of individual studies	19	Present summary statistics and effect estimates for each study or outcome.	Figure 2; extracted effect-size dataset
RESULTS: Results of syntheses	20a	Summarise characteristics and risk of bias among contributing studies.	Results: Study characteristics; Risk of bias
RESULTS: Results of syntheses	20b	Present results of all statistical syntheses, including summary estimate, precision, heterogeneity, and direction of effect.	Results: Overall exercise performance; Figures 2-4
RESULTS: Results of syntheses	20c	Present investigations of possible causes of heterogeneity.	Results: Moderator analyses; Figure 3
RESULTS: Results of syntheses	20d	Present results of all sensitivity analyses.	Results: Sensitivity analyses; Figure 6; File S6
RESULTS: Reporting biases	21	Present assessments of risk of bias due to missing results.	Results: Risk of bias, publication bias, and certainty of evidence; File S3
RESULTS: Certainty of evidence	22	Present assessments of certainty in the body of evidence.	Results: Risk of bias, publication bias, and certainty of evidence; File S5
DISCUSSION	23a	Provide a general interpretation of the results in the context of other evidence.	Discussion
DISCUSSION	23b	Discuss limitations of the evidence included in the review.	Discussion: limitations
DISCUSSION	23c	Discuss limitations of the review processes used.	Discussion: limitations
DISCUSSION	23d	Discuss implications of the results for practice, policy, and future research.	Discussion; Conclusions
OTHER INFORMATION: Registration and protocol	24a	Provide registration information for the review.	Materials and Methods: Protocol and reporting standards
OTHER INFORMATION: Registration and protocol	24b	Indicate where the review protocol can be accessed or state that no protocol was prepared.	Materials and Methods: Protocol and reporting standards
OTHER INFORMATION: Registration and protocol	24c	Describe and explain amendments to information provided at registration or in the protocol.	Not applicable
OTHER INFORMATION: Support	25	Describe sources of financial or non-financial support and the role of funders.	Funding statement
OTHER INFORMATION: Competing interests	26	Declare competing interests of review authors.	Conflict of interest statement
OTHER INFORMATION: Availability of data	27	Report availability of data, code, and other materials.	Data availability statement

Electronic Supplementary Material File S2

Database-specific search strategies

Search date: 1 April 2026. Searches combined ashwagandha-related terms with exercise-performance terms. No publication-year restrictions and no automated database filters were applied during database searching; English-language full-text original study status was assessed during eligibility screening.

Database/source	Search strategy
PubMed	((ashwagandha OR "Withania somnifera" OR "Indian ginseng" OR "winter cherry" OR Withania) AND ("exercise performance" OR "physical performance" OR "physical capacity" OR "exercise capacity" OR "aerobic capacity" OR "aerobic fitness" OR "cardiorespiratory fitness" OR "endurance performance" OR "anaerobic performance" OR "muscular strength" OR "muscular endurance" OR "power output" OR VO2max OR "time to exhaustion" OR "jump performance" OR "explosive performance" OR "strength performance"))
Web of Science Core Collection	TS=((ashwagandha OR "Withania somnifera" OR "Indian ginseng" OR "winter cherry" OR Withania) AND ("exercise performance" OR "physical performance" OR "physical capacity" OR "exercise capacity" OR "aerobic capacity" OR "aerobic fitness" OR "cardiorespiratory fitness" OR "endurance performance" OR "anaerobic performance" OR "muscular strength" OR "muscular endurance" OR "power output" OR VO2max OR "time to exhaustion" OR "jump performance" OR "explosive performance" OR "strength performance"))
Cochrane Library	(ashwagandha OR "Withania somnifera" OR "Indian ginseng" OR "winter cherry" OR Withania) in Title Abstract Keyword AND ("exercise performance" OR "physical performance" OR "physical capacity" OR "exercise capacity" OR "aerobic capacity" OR "aerobic fitness" OR "cardiorespiratory fitness" OR "endurance performance" OR "anaerobic performance" OR "muscular strength" OR "muscular endurance" OR "power output" OR VO2max OR "time to exhaustion" OR "jump performance" OR "explosive performance" OR "strength performance") in Title Abstract Keyword
Embase	('ashwagandha' OR 'withania somnifera' OR 'indian ginseng' OR 'winter cherry' OR withania) AND ('exercise performance' OR 'physical performance' OR 'exercise capacity' OR 'aerobic capacity' OR 'cardiorespiratory fitness' OR 'endurance performance' OR 'muscular strength' OR 'muscular endurance' OR 'power output' OR vo2max OR 'time to exhaustion' OR 'jump performance' OR 'explosive performance' OR 'strength performance')
SciELO	(ashwagandha OR "Withania somnifera" OR "Indian ginseng" OR "winter cherry" OR Withania) AND ("exercise performance" OR "physical performance" OR "physical capacity" OR "exercise capacity" OR "aerobic capacity" OR "aerobic fitness" OR "cardiorespiratory fitness" OR "endurance performance" OR "anaerobic performance" OR "muscular strength" OR "muscular endurance" OR "power output" OR VO2max OR "time to exhaustion" OR "jump performance" OR "explosive performance" OR "strength performance")
SPORTDiscus via EBSCOhost	TX ((ashwagandha OR "Withania somnifera" OR "Indian ginseng" OR "winter cherry" OR Withania) AND ("exercise performance" OR "physical performance" OR "physical capacity" OR "exercise capacity" OR "aerobic capacity" OR "aerobic fitness" OR "cardiorespiratory fitness" OR "endurance performance" OR "anaerobic performance" OR "muscular strength" OR "muscular endurance" OR "power output" OR VO2max OR "time to exhaustion" OR "jump performance" OR "explosive performance" OR "strength performance"))
Manual searching	Reference lists of included trials and relevant reviews were screened manually to identify additional eligible randomized controlled trials.

Electronic Supplementary Material File S3

Small-study effects, Egger regression, trim-and-fill diagnostics, and moderator funnel plots

Small-study effects were assessed using contour-enhanced funnel plots, Egger regression, and the trim-and-fill procedure within a conventional two-level random-effects framework. Because multiple correlated effect sizes were extracted from the same trials, these analyses should be considered exploratory diagnostics rather than definitive evidence for or against publication bias.

Diagnostic	Result	Interpretation
Contour-enhanced funnel plot	No clear unilateral missingness pattern	No obvious one-sided missing-study pattern was detected.
Egger regression	Intercept = 0.82; SE = 0.98; p = 0.406	No statistically significant evidence of small-study effects.
Trim-and-fill side selection	Left side	Default side-selection indicated the left side.
Imputed missing effect sizes	k0 = 0	No missing effect sizes were imputed by the L0 estimator.
Adjusted pooled estimate	g = 0.47, 95% CI [0.25, 0.69]	Adjusted and unadjusted estimates were essentially unchanged.

Figure S3. Small-study-effect diagnostics for the primary outcome. Panel A shows the contour-enhanced funnel plot. Panel B shows the trim-and-fill adjusted funnel plot. The blue vertical line indicates the pooled effect reported for the primary three-level model to maintain consistency with the manuscript.

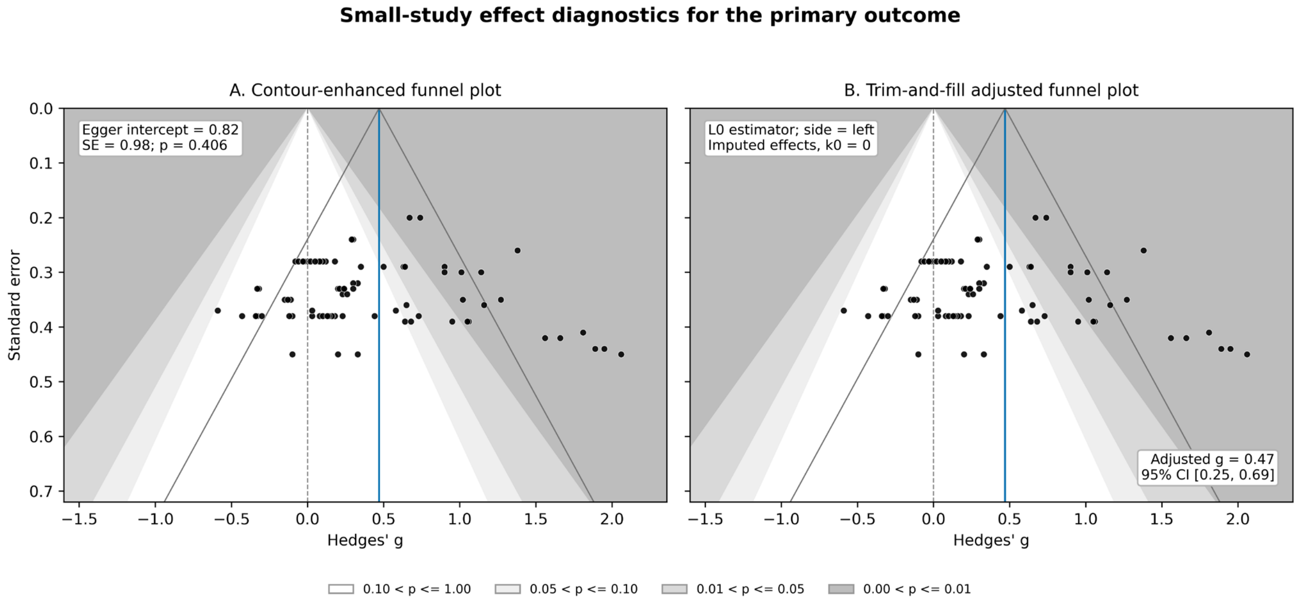

Figure S3.2. Contour-enhanced funnel plots by moderator subgroup. Each panel displays outcome-level effect sizes for the corresponding moderator subgroup. Shaded contours denote approximate two-sided significance regions, and Egger regression p values are shown within each panel. These subgroup diagnostics are exploratory because several panels contain few independent studies and multiple correlated effects from the same trials.

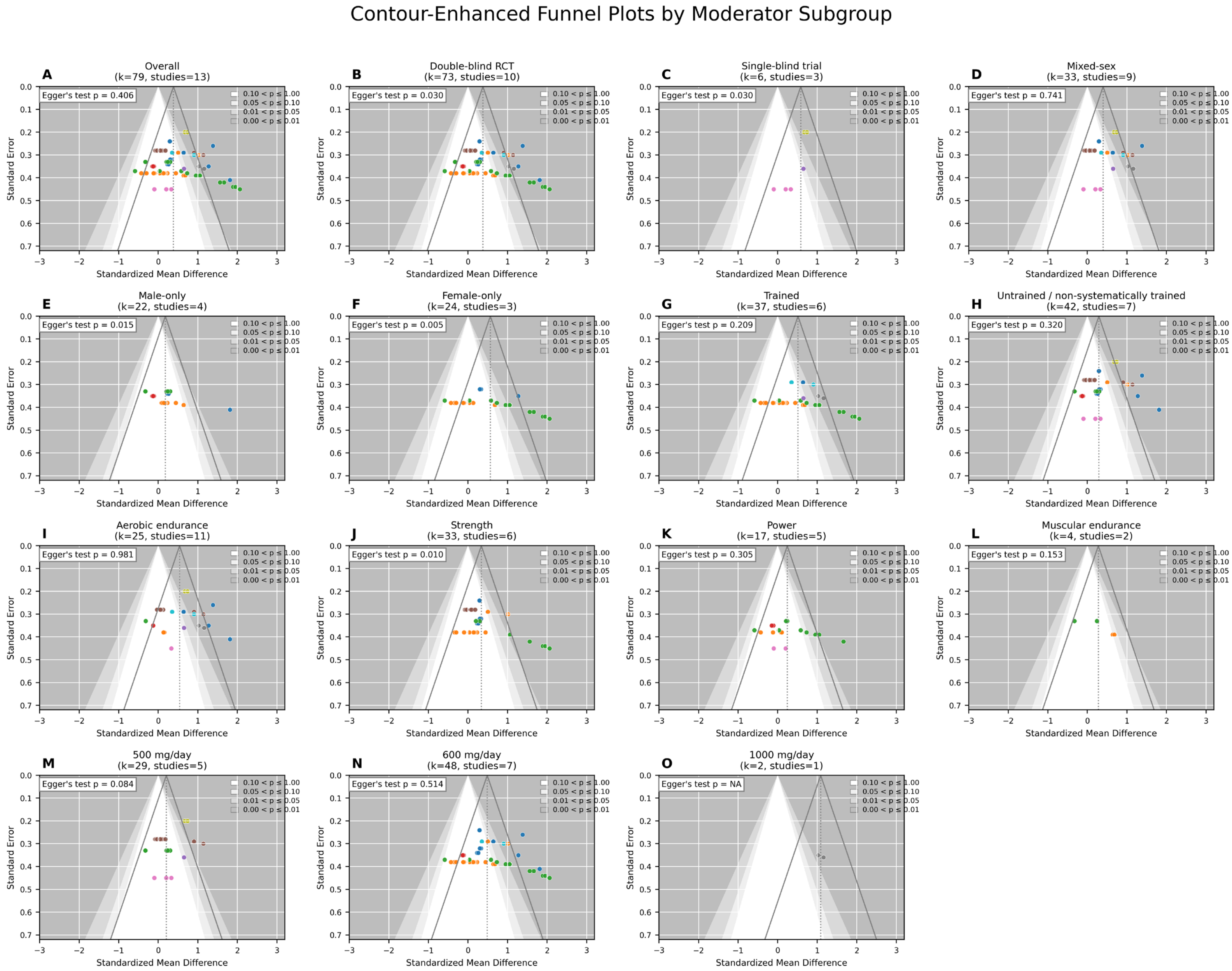

Electronic Supplementary Material File S4

Power visualization, replicability diagnostics, and moderator power plots

Observed power was calculated from the absolute standard normal deviate for each outcome-level effect size using a two-sided alpha of 0.05. The R-index was calculated as 2 x median observed power minus the observed success rate.

Metric	Value
Median observed statistical power	14.0%
Success rate	26.6%
R-index	1.4%

Figure S4. Power visualization and replicability diagnostics for the included outcome-level effect sizes.

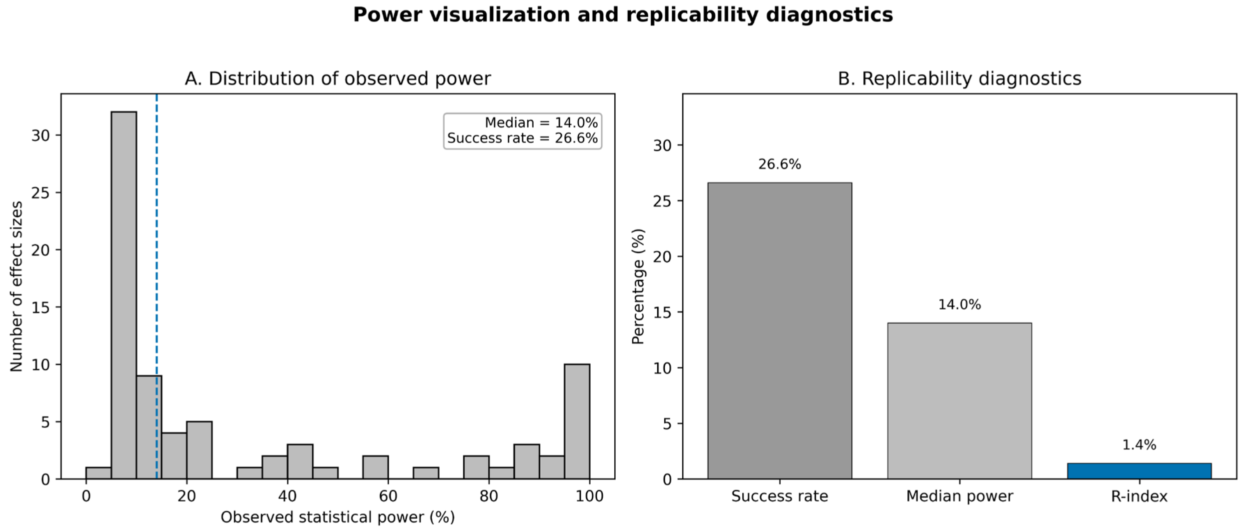

Figure S4.2. Power visualization by moderator subgroup. Background bands represent approximate observed-power regions across the standard-error scale; points represent observed outcome-level effect sizes. The solid vertical line indicates the subgroup-specific pooled effect, and the dashed triangular boundaries provide reference limits. Median observed power and R-index values are shown below each panel.

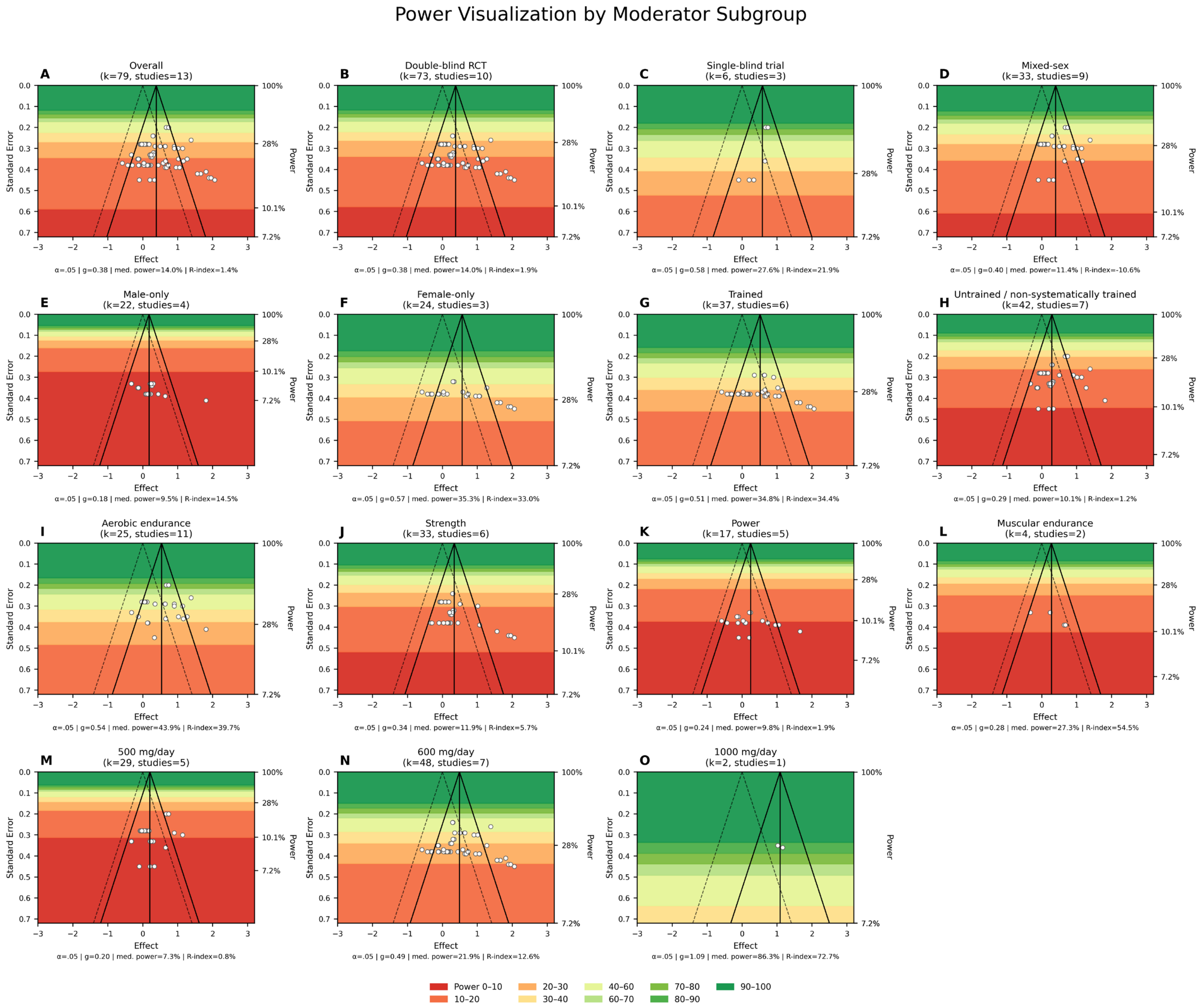

Electronic Supplementary Material File S5

GRADE certainty-of-evidence assessment

Certainty was assessed across risk of bias, inconsistency, indirectness, imprecision, and publication bias/small-study effects. Randomized evidence started at high certainty and was downgraded according to the judgments shown below.

Table S5.1. Summary evidence profile by moderator subgroup.

Domain	Subgroup	Studies	Effects	Effect estimate	p	P_between	GRADE	Certainty
Overall	All included effects	13	79	g = 0.47, 95% CI [0.25, 0.69]	NA	NA	⊕⊕⊕◯	Moderate
Study design	Double-blind RCT	10	73	g = 0.46, 95% CI [0.20, 0.73]	<0.001	0.91	⊕⊕⊕◯	Moderate
Study design	Single-blind trial	3	6	g = 0.52, 95% CI [0.04, 1.01]	0.039	0.91	⊕⊕◯◯	Low
Sex	Mixed-sex	9	33	g = 0.56, 95% CI [0.33, 0.78]	<0.001	0.672	⊕⊕◯◯	Low
Sex	Female-only	3	24	g = 0.52, 95% CI [-0.20, 1.25]	0.149	0.672	⊕⊕◯◯	Low
Sex	Male-only	4	22	g = 0.20, 95% CI [-0.10, 0.49]	0.183	0.672	⊕⊕◯◯	Low
Training status	Trained	6	37	g = 0.65, 95% CI [0.27, 1.03]	0.002	0.156	⊕⊕⊕◯	Moderate
Training status	Untrained or non-systematically trained	7	42	g = 0.33, 95% CI [0.07, 0.59]	0.015	0.156	⊕⊕⊕◯	Moderate
Exercise type	Aerobic endurance	11	25	g = 0.54, 95% CI [0.22, 0.85]	0.002	0.006	⊕⊕⊕◯	Moderate
Exercise type	Strength	6	33	g = 0.48, 95% CI [-0.04, 1.01]	0.068	0.006	⊕⊕⊕◯	Moderate
Exercise type	Power	5	17	g = 0.18, 95% CI [-0.18, 0.54]	Not significant	0.006	⊕⊕◯◯	Low
Exercise type	Muscular endurance	2	4	g = 0.29, 95% CI [-0.83, 1.41]	0.303	0.006	⊕◯◯◯	Very low
Dosage	500 mg/day	5	29	g = 0.31, 95% CI [0.02, 0.59]	0.038	0.224	⊕⊕⊕◯	Moderate
Dosage	600 mg/day	7	48	g = 0.52, 95% CI [0.18, 0.85]	0.003	0.224	⊕⊕⊕◯	Moderate
Dosage	1000 mg/day	1	2	g = 0.95; 95% CI not estimable from the available model output	0.144	0.224	⊕◯◯◯	Very low

Table S5.2. Detailed GRADE domain judgments.

Domain	Subgroup	Risk of bias	Inconsistency	Indirectness	Imprecision	Publication bias / small-study effects
Overall	All included effects	Not serious	Serious (-1)	Not serious	Not serious	Not serious; cannot be fully excluded
Study design	Double-blind RCT	Not serious	Serious (-1)	Not serious	Not serious	Not serious; some small-study concern
Study design	Single-blind trial	Serious (-1)	Not serious / unclear	Not serious	Serious (-1)	Undetected; sparse evidence
Sex	Mixed-sex	Not serious	Serious (-1)	Serious (-1)	Not serious	Not serious; cannot be fully excluded
Sex	Female-only	Not serious / some concern	Serious (-1)	Not serious	Serious (-1)	Undetected; few independent studies
Sex	Male-only	Not serious / some concern	Not serious / unclear	Not serious	Serious (-1)	Serious concern due to sparse sex-specific evidence (-1)
Training status	Trained	Not serious	Serious (-1)	Not serious	Not serious	Not serious; cannot be fully excluded
Training status	Untrained or non-systematically trained	Not serious	Serious (-1)	Not serious	Not serious / borderline	Not serious; cannot be fully excluded
Exercise type	Aerobic endurance	Not serious	Serious (-1)	Not serious	Not serious	Not serious
Exercise type	Strength	Not serious	Not serious / some concern	Not serious	Serious (-1)	Not serious; cannot be fully excluded
Exercise type	Power	Not serious / some concern	Serious (-1)	Not serious	Serious (-1)	Undetected; few independent studies
Exercise type	Muscular endurance	Not serious / unclear	Serious (-1)	Not serious / some concern	Very serious (-2)	Serious concern due to very sparse evidence (-1)
Dosage	500 mg/day	Not serious	Not serious / some concern	Not serious	Serious (-1)	Not serious; cannot be fully excluded
Dosage	600 mg/day	Not serious	Serious (-1)	Not serious	Not serious	Not serious; cannot be fully excluded
Dosage	1000 mg/day	Not serious / unclear	Not estimable	Not serious / some concern	Very serious (-2)	Serious concern due to one study / two effects (-1)

Downgrading rationale notes. Overall and most moderate-certainty subgroups were primarily downgraded for inconsistency or residual uncertainty from heterogeneity. Low- or very-low-certainty subgroups were downgraded for sparse evidence, few independent studies, wide confidence intervals, and/or publication-bias concerns that could not be excluded. The 1000 mg/day and muscular-endurance subgroups were particularly limited by very few contributing effects.

Abbreviations: CI, confidence interval; GRADE, Grading of Recommendations Assessment, Development and Evaluation; RCT, randomized controlled trial. GRADE symbols: ⊕⊕⊕◯ = moderate certainty; ⊕⊕◯◯ = low certainty; ⊕◯◯◯ = very low certainty.

Electronic Supplementary Material File S6

Leave-one-out sensitivity analyses at effect-size and study levels

Leave-one-out analyses were conducted by omitting one outcome-level effect size at a time (Level 2) and one study at a time (Level 3). The primary pooled effect remained positive and statistically significant across all omissions, indicating that the overall finding was not driven by a single effect size or a single trial.

Leave-one-out sensitivity analysis based on three-level random-effects REML model
Overall: g = 0.466, 95% CI [0.246, 0.686], p = 3.409e-05, I2 = 63.1%
Level 2 range: g = 0.448 to 0.485; I2 = 60.4% to 64.1%
Level 3 range: g = 0.412 to 0.509; I2 = 53.6% to 67.4%

Figure S6.1. Sensitivity analysis based on leave-one-out at the effect-size level (Level 2), part 1.

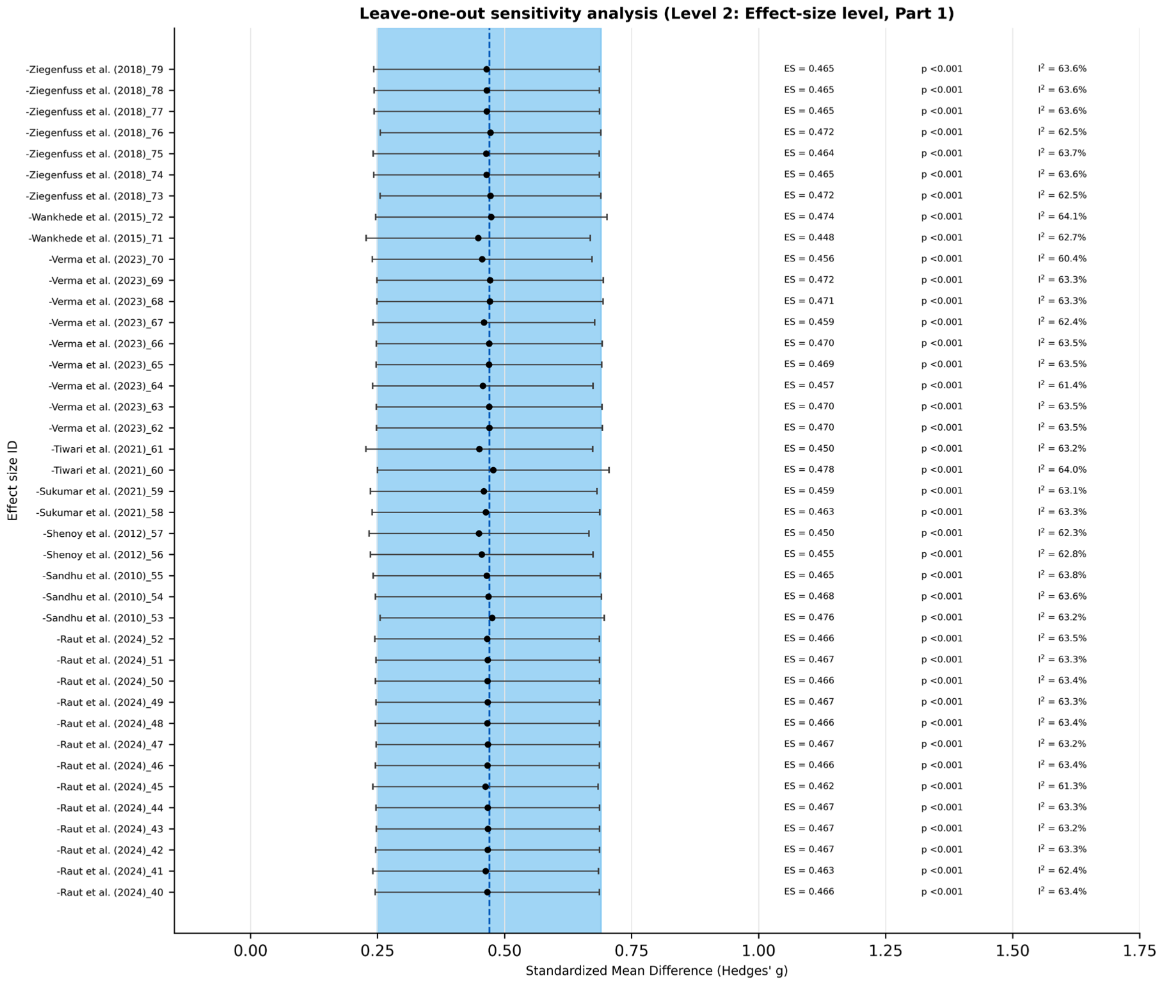

Figure S6.2. Sensitivity analysis based on leave-one-out at the effect-size level (Level 2), part 2.

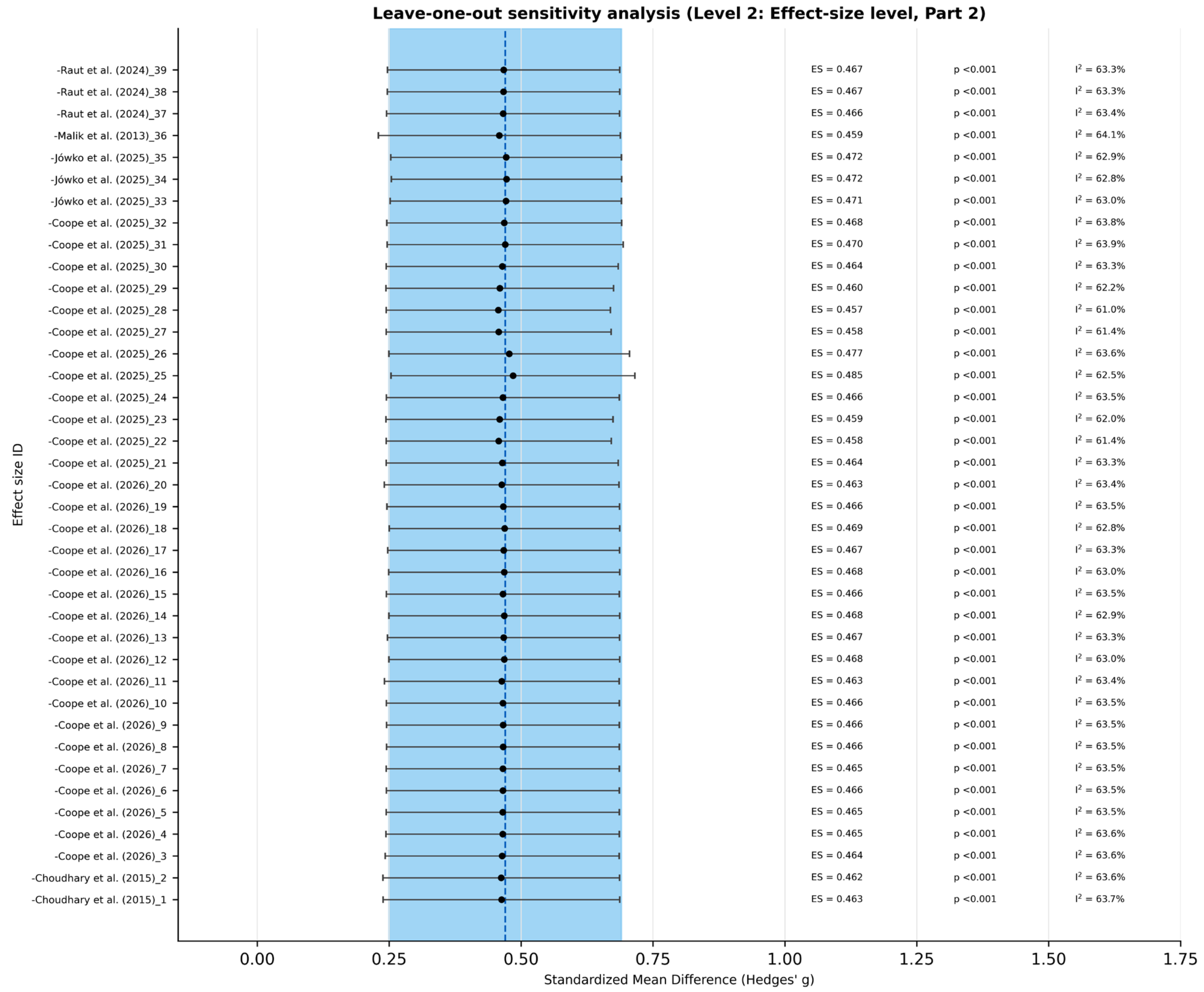

Figure S6.3. Sensitivity analysis based on leave-one-out at the study level (Level 3).

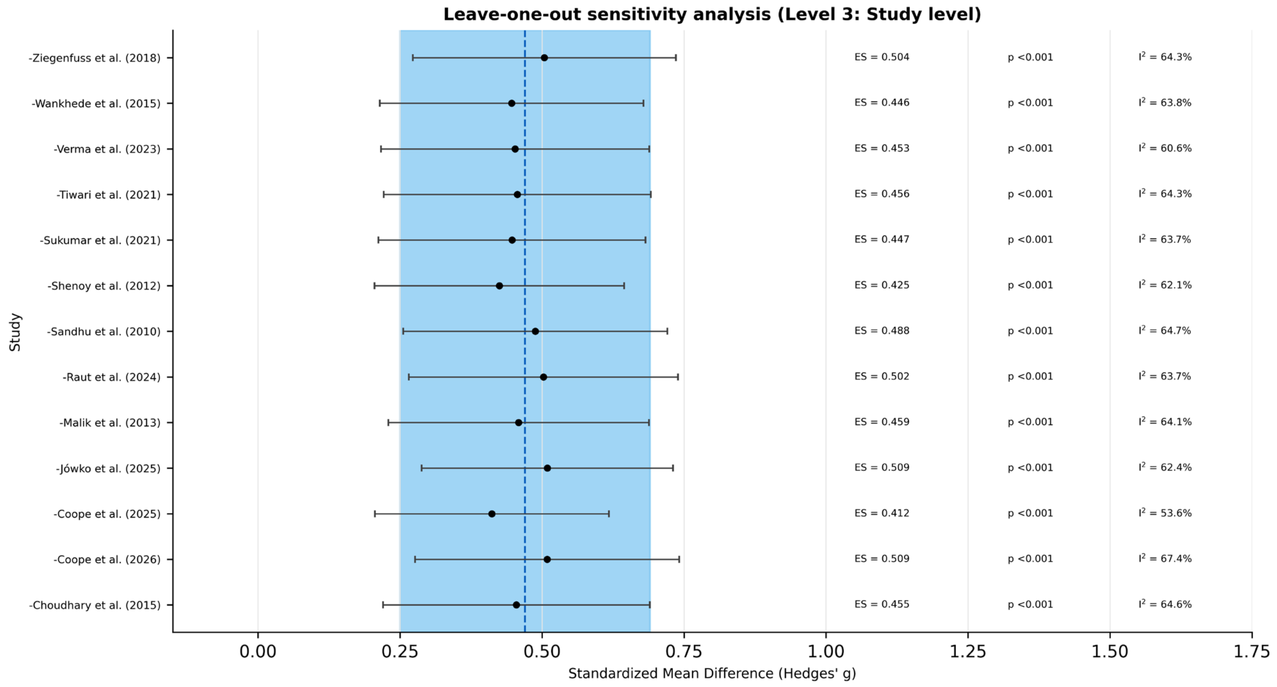

Supplement: Supplementary file 1 [file nutrients-18-01915-s001.zip › nutrients-4336921-supplementary.pdf]
